# First Detection of *Bartonella* spp. in Small Mammals from Rice Storage and Processing Facilities in Myanmar and Sri Lanka

**DOI:** 10.3390/microorganisms9030658

**Published:** 2021-03-22

**Authors:** Inga Böge, Martin Pfeffer, Nyo M. Htwe, Pyai P. Maw, Siriwardana Rampalage Sarathchandra, Vincent Sluydts, Anna P. Piscitelli, Jens Jacob, Anna Obiegala

**Affiliations:** 1 Institute of Animal Hygiene and Veterinary Public Health, University of Leipzig, 04103 Leipzig, Germany; inga.boege@web.de (I.B.); pfeffer@vetmed.uni-leipzig.de (M.P.); 2 Department of Agriculture, Plant Protection Division, Bayintnaug Rd, Gyogone, Insein Township, Yangon 11011, Myanmar; nyomehtwe@gmail.com (N.M.H.); pyaiphyo2009@gmail.com (P.P.M.); 3 Rice Research and Development Institute, Batalagoda, Ibbagamuwa, Kurunegala 60000, Sri Lanka; siriwardanadoa@gmail.com; 4 Department of Biology, University of Antwerp, 2610 Wilrijk, Belgium; vincent.sluydts@uantwerpen.be (V.S.); annapia.piscitelli@uantwerpen.be (A.P.P.); 5 Julius Kühn-Institute, Federal Research Institute for Cultivated Plants, Institute for Plant Protection in Horticulture and Forests, Vertebrate Research, 48161 Münster, Germany; jens.jacob@julius-kuehn.de

**Keywords:** *Bartonella kosoyi*, *Bartonella henselae*, *Bartonella* sp. SE-Bart-D, zoonosis, rodents, Bandicoot rats, *Rattus* spp., *Suncus murinus*, *Mus booduga*

## Abstract

(1) Background: *Bartonella* spp. are zoonotic bacteria with small mammals as main reservoirs. *Bartonella* spp. prevalence in small mammals from Myanmar and Sri Lanka are yet unknown. (2) Methods: Small mammals were snap trapped in Sri Lanka and Myanmar in urban surroundings. Spleens-derived DNA was screened for *Bartonella* spp. using conventional PCR based on three target genes. Positive samples were sequenced. (3) Results: 994 small mammals were collected comprising 6 species: *Bandicota bengalensis*, *Bandicota indica*, *Rattus exulans*, *Rattus rattus, Mus booduga,* and *Suncus murinus*. In Myanmar, the *Bartonella* prevalence in Bandicoot rats (68.47%) was higher than in *Rattus rattus* (41.67%), *Rattus exulans* (21.33%), and *Suncus murinus* (3.64%). Furthermore the prevalence in Myanmar (34%, *n* = 495) was twice as high as in Sri Lanka (16%, *n* = 499). In Sri Lanka, *Bartonella* spp. occurred almost exclusively in *R. rattus*. In Myanmar, *Bartonella kosoyi* was mainly detected (56%), followed by *Bartonella* sp. KM2529 (15%), *Bartonella* sp. SE-Bart D (12%) and *Bartonella henselae* (1%). In Sri Lanka, *B. phoceensis* (60%) and *Bartonella* sp. KM2581 (33%) were predominant. (4) Conclusions: *Bartonella* spp. were detected in all investigated small mammal species from Myanmar and Sri Lanka for the first time. *Bartonella kosoyi* and *B. henselae* are zoonotic. As these small mammals originated from urban settlements, human bartonellosis seems likely to occur.

## 1. Introduction

The genus Bartonella includes over 40 species and subspecies [1] of gram-negative, hemotropic, facultative intracellular bacteria that infect endothelial cells and erythrocytes of mammalian hosts. *Bartonella* spp. are vector-borne and commonly transmitted via blood-sucking arthropods. Rodents are known to be the main reservoir of different *Bartonella* spp. with fleas the main vector within and between rodent populations [2,3]. Among the variety of zoonotic Bartonellae, *Bartonella elizabethae, Bartonella grahamii*, *B. henselae, Bartonella tribocorum,* and *Bartonella vinsonii* subsp. *arupensis* are described in association with rodent reservoirs [2,4,5].

*Bartonella* spp. infections are associated with various human diseases such as cat-scratch disease (*Bartonella henselae*), trench fever (*Bartonella quintana*), and Oroya fever (*Bartonella bacilliformis*). Cases of bartonellosis associated with rodents are described from all over the world with clinical symptoms such as fever, muscle and joint pain, neuroretinitis, and endocarditis [6,7]. Regarding Asia, unspecific clinical symptoms are caused by *B. henselae* in human patients from Laos and Taiwan [8,9]. Further, *Bartonella tamiae* and other *Bartonella* spp. were found in febrile patients from Thailand [10]. Until now no cases of bartonellosis in humans have been reported from Sri Lanka and Myanmar. Due to the unspecific symptoms, and missing diagnostic tools, infections could have been overlooked.

Nonetheless, rodent-associated zoonotic Bartonellae can pose a risk in countries such as Myanmar and Sri Lanka where close contact between humans and rodents is common [5,11]. Myanmar covers an area of 676,552 km^2^ and population size is 54 million people [12] of which 70% live in rural areas and work in the agricultural sector achieving 30% of the gross domestic product [13,14]. About 21 million people live in Sri Lanka in an area of about 65,705 km^2^ [15]. As of 2018, 25.7% of the employed population works in the agricultural sector [16].

The genera Bandicota, Rattus and Mus include synanthropic pest rodent species that live in close contact with humans in those countries [17]. In other Asian countries many newly discovered Bartonella species and high Bartonella prevalence was detected in these rodents [2]. There are a few studies of Bartonella infections indicating prevalence ranging from 9 to 68% in urban rodents in Southeast Asia [18,19,20,21,22,23,24]. In comparison, little is known about the percentage of Bartonella-positive rodents in rural areas. Published reports are restricted to a study that considered Bartonella prevalence in small mammals across a gradient of human density that included five agricultural sites in Laos (11.9%) and Thailand (11.0%) [25].

In Myanmar, a previous study [26] detected *Bartonella* spp. prevalence in fleas associated with cats and rodents in an area along the Thai-Myanmar border. Six out of 90 fleas were tested positive for Bartonella DNA and a newly described Bartonella species was detected from one flea collected from a *Rattus surifer* [26]. In Sri Lanka, a study analyzed *Bartonella* spp. infections in dogs with positive individuals showing even multiple infections [27].

To date, no study of Bartonella prevalence in small mammals as possible reservoir hosts has been conducted in Myanmar and Sri Lanka. Because of the close contact between rodents and humans in various parts of these countries, it is important to study rodent-associated *Bartonella* spp. to assess the risk for human health and companion animals.

Thus, the main aim of this study was to detect small mammal species living close to human settlements in Myanmar and Sri Lanka and to evaluate the Bartonella prevalence in those small mammal species for the first time. Another purpose was to identify the Bartonella species in these samples by molecular techniques to evaluate their zoonotic potential.

## 2. Materials and Methods

### 2.1. Study Sites

**Figure 1:**
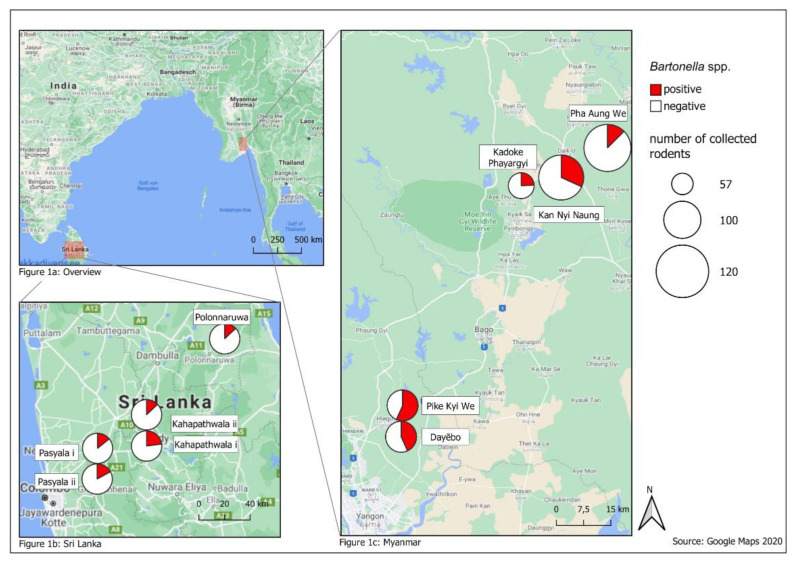
Study sites shown in overview (Figure 1a) and in detail in Sri Lanka (Figure 1b) and Myanmar (Figure 1c): Distribution of *Bartonella* spp. DNA positive small mammals. (Maps: QGIS 3.2.1 “Bonn”, Open Source Geospatial Foundation 2019).

Small mammals were trapped at five sites in Myanmar and Sri Lanka in the years 2018 and 2019. Traps were set in villages at structures used for rice storage and rice processing in Myanmar and Sri Lanka (Figure 1).

In Myanmar, study sites were located in granaries or mills in the rural delta and coastal zone, where the main agricultural production is located. Dayēbo (17°6′19.548″ N; 96°14′44.124″ E) and Pike Kye We (17°4′58.926″ N; 96°0′0″ E) are rural areas mainly characterized by rice production [28]. At the sites Kan Nyi Naung (17°6′50.036″ N; 96°43′40.404″ E), Kadoke Phayargyi (17°41′20.188″ N; 96°35′30.933″ E) and Pha Aung We (17°46′18.581″ N; 96°41′10.046″ E) subsistence agriculture prevails [29]. The climatological conditions in Myanmar are divided in a dry season (November–May) and a wet season (June–October) [30].

Small mammal trapping in Sri Lanka was conducted in Pasyala (Gampaha District) i (7°9′0.742″ N; 80°8′14.006″ E), Pasyala (Gampaha District) ii (7°9′12.085″ N; 80°8′4.459″ E), Kahapathwala (Kurunegala District) i (7°23′34.674″ N; 80°27′52.981″ E) and Kahapathwala (Kurunegala District) ii (7°23′43.318″ N; 80°28′27.04″ E) that are characterized by an increase in housing units indicating population growth in these areas. Sinhapura (Polonnaruwa District) i (8°1′17.976″ N; 81°1′19.091″ E) is characterized by rural agriculture with 39.1% employment in this sector [31,32,33]. There is a dry (minor) season (April–August) and a wet (major) season (October–February) [34].

### 2.2. Sampling of Small Mammals, Preparation of Samples and DNA Extraction

Small mammals were obtained and killed by snap trapping as part of regular pest rodent control operations. In the countries where the work was conducted, snap trapping is not regarded as animal experimentation. Hence, no animal ethics permits were required. In Myanmar, small mammals were trapped from June to December in 2018 and from May to December in 2019. In each site, 10 snap traps (Kness, big-snap-E rat trap) and 5 local-made bamboo traps were baited with unhulled rice and set opportunistically in and around 10 small-holder rice storage facilities for 450 trap nights per site and year (total trap nights; 4500). In Sri Lanka, small mammals were captured in two trapping sessions in June–December 2018 and two trapping sessions in March–August 2019 (for details see [35]). In each of the five sites, 10 snap traps (Kness, big-snap-E rat trap) were baited with unhulled rice and roasted coconut and set for three consecutive nights per session opportunistically in and around ten small-holder rice storage or rice processing facilities resulting in 600 trap nights per site and year (total trap nights: 6000).

Trapped small mammals were identified to species level based on morphology [17] supplemented by phylogenetic analysis of a partial fragment of the *cytochrome b* gene [35]. Standard body measurements were recorded, and several organ samples were collected including the spleen. Spleen samples were stored in 70% ethanol until processing and analyses for *Bartonella* spp.

Before processing the spleen samples for DNA extraction, the ethanol was washed out with phosphate-buffered-saline (PBS, pH 7.2) by transferring in PBS and incubating for four hours and drying afterwards. Then, all samples were homogenized with 0.6 g sterile ceramic beads (sized 1.4 mm, Peqlab Biotechnologie, Erlangen, Germany) four times the sample’s weight with PBS (pH 7.2). The Precellys^®^ 24 tissue homogenizer (Bertin Technologies, Montigny Le Bretonneux, France) was used for mechanical disruption with 5500 rpm for 2 × 15 s with a 10 s break between both runs.

Afterwards the DNA was extracted using the QIAamp DNA Mini Kit (Qiagen, Hilden, Germany) according to the manufacturer’s instructions. The DNA samples were measured qualitatively and quantitatively with the NanoDrop ND-1000 (PeqLab Biotechnologie GmbH). Samples with a DNA concentration >40 ng/μL were diluted with the kit’s elution buffer to receive a DNA concentration of 20–40 ng/μL for each sample.

### 2.3. Detection of Bartonella spp. and Sequence Analysis

The presence of *Bartonella* spp. was detected in DNA samples by conventional polymerase chain reaction (PCR) targeting the NADH dehydrogenase subunit (*nuoG*) with an amplicon size of 346 base pairs (bp). Additionally, all samples were further analyzed in two PCRs targeting the *gltA* gene (378 bp) and a fragment of the 16S-23S rRNA ITS region (453–780 bp) [36,37,38,39].

The amplicon of the *nuoG* was detected with previously published PCR protocols and the genus specific primers nuoGF (5′-GGCGTGATTGTTCTCGTTA-3′) and nuoGR (5′-CACGACCACGGCTATCAAT-3′) [39]. For the detection of *Bartonella* spp. DNA based on the *gltA* gene the genus specific primers BhCS.781p (5′-GGGGACCAGCTCATGGTGG-3′) and BhCS.1137n (5′-AATGCAAAAAGAACAGTAAACA-3′) were used. The amplification consisted of 45 cycles of denaturation at 95 °C for 30 s, annealing at 53 °C for 30 s and elongation at 72 °C for 1 min [36,38]. The amplification of the 16S-23S rRNA ITS region was performed with forward (Ba325s: 5′-CTTCAGATGATGATCCCAAGCCTTCTGGCG-3′) and reverse primers (Ba1100as: 5′-GAACCGACGACCCCCTGCTTGCAAAGC-3′) as previously published, after minimal modifications in the set-up of the amplification run: 40 cycles for 30 s at 94 °C, for 30 s at 66 °C, for 50 s at 72 °C [37].

PCR products were prepared with DNA Gel Loading Dye (6×) (Thermo Fisher Scientific Baltics UAB, Vilnius, Lithuania) for gel electrophoresis in 2% agarose. The results were visualized by UV light using the UVP GelSolo streamlined gel documentation (Analytik Jena AG, Jena, Germany). Afterwards 125 positive samples were selected for sequencing to cover all small mammal species detected at each location in Myanmar and Sri Lanka in both seasons of each collection year. The samples were purified for sequencing using the NucleoSpin Gel and PCR clean-up kit (Macherey-Nagel GmbH & Co. KG, Düren, Germany) as recommended by the manufacturer. Further, samples were sequenced commercially with forward and reverse primers of the 16S-23S rRNA ITS region (Interdisziplinäres Zentrum für Klinische Forschung, Leipzig, Germany). The sequences were trimmed using Bionumerics v.7.6.1. (Applied Maths Inc., Austin, TX, USA) and compared to available data in GenBank with BLASTn (https://blast.ncbi.nlm.nih.gov/Blast.cgi (accessed on 25 February 2021)) [40]. Obtained sequences were uploaded in GenBank under the following accession numbers (MW194932-MW194978, MW222168-MW222177, MW233783-MW233857).

### 2.4. Statistical Analysis

Confidence intervals (95% CI) for the prevalence of *Bartonella* spp. in small mammals were determined by the Clopper and Pearson method [41] with setting the level of alpha to 0.05 using GraphPad Prism Software v. 4.0 (GraphPad Software Inc., San Diego, CA, USA).

The independence of compared prevalence rates concerning small mammal species, age, sex, trapping year, season, and in Myanmar additionally regarding the habitat, was tested using Pearson’s Chi-squared test. Tests with two variables were considered to be significant if *P* (probability) < 0.05. Only samples tested positive for all three target genes were considered positive for *Bartonella* spp.

## 3. Results

### 3.1. Captured Small Mammal Species

In Myanmar, 495 small mammals were trapped belonging to five species: *Bandicota bengalensis* (n_2018_ = 94; n_2019_ = 61); *B. indica* (n_2018_ = 6; n_2019_ = 2); *Rattus exulans* (n_2018_ = 59; n_2019_ = 91); *R. rattus* (n_2018_ = 51; n_2019_ = 21); *Suncus murinus* (n_2018_ = 36; n_2019_ = 74) (Table 1). Of these small mammals, 329 were caught at mills (*B. bengalensis*: 149, *B. indica*: 8, *R. exulans*: 67, *R. rattus:* 22, *S. murinus:* 83) and 166 at granaries (*B. bengalensis*: 6, *R. exulans*: 83, *R. rattus*: 50, *S. murinus*: 27). In Sri Lanka, 499 small mammals were captured representing six species: *B. bengalensis* (n_2019_ = 1); *B. indica* (n_2018_ = 5; n_2019_ = 8); *Mus booduga* (n_2018_ = 4); *R. exulans* (n_2018_ = 2; n_2019_ = 3); *R. rattus* (n_2018_ = 206; n_2019_ = 227); *S. murinus* (n_2018_ = 33; n_2019_ = 10) (Table 2). All small mammals from Sri Lanka were caught at village stores.

### 3.2. Bartonella spp. Prevalence in Small Mammals

Overall 248 out of 994 samples (25%; 95% CI: 22.3–27.8%) tested positive for *Bartonella* spp. DNA by conventional PCR targeting all three target genes altogether (Table 3). Altogether 168 out of 495 (34%; 95% CI: 29.8–38.3%) small mammals in Myanmar and 80 out of 499 (16%; 95% CI: 12.9–19.6%) small mammals in Sri Lanka were positive. The prevalence in Myanmar was significantly higher compared to Sri Lanka (χ^2^ = 42.556, df = 1, *P* < 0.0001).

In Myanmar, the prevalence ranged in 2018 from 7% in Pha Aung We to 46% in Pike Kye We and in 2019 from 15% in Pha Aung We to 70% in Pike Kye We (Figure 1). The prevalence of the adjacent sites Pike Kye We and Dayēbo were higher than at Kadoke Phayargyi, Pha Aung We and Kan Nyi Naung (χ^2^ = 41.273, df = 1, *P* < 0.0001). In contrast, Bartonella prevalence in Sri Lanka was similar between years (10–28%) and sites (10–28%) (Figure 1).

In Myanmar, prevalence was higher in *Bandicota* than in *Rattus* spp. and *S. murinus* (χ^2^ = 88.894, df = 1, *P* < 0.0001). The prevalence of Bartonella in the genus *Rattus* was significantly higher compared to *S. murinus* (χ^2^ = 27.250, df = 1, *P* < 0.0001) in Myanmar. Likewise, in Sri Lanka the prevalence in *R. rattus* was higher than in all other small mammal species which were caught by lower numbers (χ^2^ = 10.697, df = 1, *P* = 0.0011).

No significant difference in the prevalence of *Bartonella* spp. in Myanmar was detected between males and females (χ^2^ = 0.253, df = 1, *P* = 0.6153), between the years 2018 and 2019 (χ^2^ = 1.527, df = 1, *P* = 0.2165) and the rainy and dry season (χ^2^ = 0.005, df = 1, *P* = 0.9429). In Myanmar, the prevalence of Bartonella in small mammals caught in granaries was significantly higher than in small mammals caught in mills (χ^2^ = 5.198, df = 1, *P* = 0.0226). However, due to the unequal distribution of small mammal species in granaries and mills, these results may be regarded as slightly distorting. The prevalence of adult small mammals was significantly higher compared to the lower prevalence of sub-adult small mammals (χ^2^ = 54.035, df = 1, *P* < 0.0001). There was no significant difference in Sri Lanka for the Bartonella prevalence between adults and sub-adults (χ^2^ = 0.028, df = 1, *P* = 0.8677), males and females (χ^2^ = 0.076, df = 1, *P* = 0.7826), the years 2018 and 2019 (χ^2^ = 0.000, df = 1, *P* = 0.9844), and the seasons (χ^2^ = 0.005, df = 1, *P* = 0.9443) [34] (Supplementary Tables S1 and S2).

Several Bartonella co-infections were detected in small mammals from Myanmar and Sri Lanka. In Myanmar 30 out of 168 positively tested small mammals (18%, 95% CI: 12.4–24.5%) with double infections were detected by PCR targeting the ITS region. Double infections in Myanmar were most common in *B. bengalensis* (21/96; 22%, 95% CI: 14.1–31.5%) and *R. rattus* (7/30; 23%, 95% CI: 9.9–42.3%) and at the sites Dayēbo (12/45; 27%, 95% CI: 14.6–41.9%) and Kadoke Phayargyi (4/14; 29%, 95% CI: 8.4–58.1%). In Sri Lanka, 11 out of 80 positively tested samples (14%, 95% CI: 7.1–23.3) showed double infections. Here, co-infections were detected only in one rodent species, *R. rattus,* caught at the sites Pasyala and Kahapathwala.

### 3.3. Sequence Analysis of Bartonella-Positive Samples

Sequence analyses were performed for 125 *Bartonella*-positive samples based on the ITS region (53%) and 11 different Bartonella strains were detected (Table 4). In Myanmar, the predominantly detected species was *B. kosoyi* (41/73; 56%, 95% CI: 44.1–67.8%). *Bartonella* sp. KM2529 (11/73; 15%, 95% CI: 7.8–25.4%) and *Bartonella* sp. SE-Bart-D (9/73; 1%, 95% CI: 5.8–22.1%) were detected in small mammals from Myanmar as well. The most frequently identified *Bartonella* species in Sri Lanka was *B. phoceensis* (25/42; 60%, 95% 95% CI: 43.3–74.4%) followed by *Bartonella* spp. KM2581 (14/42; 33%, 95% CI: 19.6–49.6%). The detected *Bartonella* spp. were evenly distributed and no effect of small mammal species, age, season and habitat was apparent. However, *B. kosoyi*. was exclusively detected in *B. indica* from Myanmar.

Two different *Bartonella* spp., (Accession Number: EF407566) and (Acc. No.: EF213776) were detected in one *R. exulans* caught in Kan Nyi Naung, Myanmar. Two *R. norvegicus* from China were earlier positive for *Bartonella* sp. RN25BJ, respectively, *Bartonella* sp. RN28BJ. Furthermore, 10 samples showed <97% identity with sequences in GenBank, but 98–100% within group-similarity and can be assembled in groups with >98% agreement (Table 4). Group 1 consists of three samples from Myanmar and showed high similarities to *Bartonella* sp. KM 2529. Group 2 consists of four samples from *R. rattus* caught at two different sites (Pasyala and Kahapathwala) in Sri Lanka which are most similar to *Bartonella* sp. SE-Bart-D. Three samples from *S. murinus* caught in 2019 at two different sites in Myanmar form Group 3 and show high similarity to an uncultured *Bartonella* clone 5199 (Table 4). Seven samples were not considered Bartonella-positive as they yielded only a similarity <92% with other sequences from the current study and sequences in GenBank.

## 4. Discussion

This study presents the first detection of *Bartonella* spp. in small mammals from Myanmar and Sri Lanka suggesting their potential as reservoirs. In this study, up to five different small mammal species occurred sympatrically in Myanmar and Sri Lanka. The two genera *Bandicota* and *Rattus* caught in this study belong to the major agricultural pest species in these countries [17]. Moreover, the invasive species *S. murinus* which has its origin in India was found in this study [42]. *Mus booduga* which was also detected is known as field mouse and not as true commensal in India, Sri Lanka, and Nepal [17]. Rodents, especially of the species *R. rattus* and *R. exulans* occur mainly in villages and less in cropping areas, however, *R. rattus* is also known for causing significant damage in agriculture in South Asia [17]. Further, Bandicoot rats are reported from all sorts of human dwellings, but especially *B. bengalensis* is known as a major pest. The population density of *B. indica* is lower compared to other rodent species and is identified as minor pest species [17]. In Myanmar and Sri Lanka, the mentioned small mammal species are moreover known as reservoirs of zoonotic pathogens such as the Hantaan virus and *Leptospira* spp. [43,44,45] which adds relevance to the livelihoods of people beyond their importance as considerable pest rodents [17]. The inevitable proximity of humans and rodents [46] is likely to increase the risk of transmission of zoonotic pathogens from rodents to humans. This is confirmed by high antibody titers of the Hantaan virus in rodents and in humans living in close contact with rodents in Thailand and Taiwan [47,48]. The commensal shrew *S. murinus* is a reservoir for *Yersinia pestis* and responsible for recent human plague outbreaks in Vietnam [49]. In Sri Lanka, *Y. pestis* was isolated from Bandicoot rats, but reports about plague hosts in Myanmar are missing [50].

Especially in Southeast Asia rodent-associated infectious diseases are emerging [51]. The *Bartonella* spp. clade associated with rodents shows a wide disparity regarding *Bartonella* species and potential hosts [52]. Several studies showed prevalence rates ranging from 9 to 67% in urban rodents from Thailand (55.6–67.6% [18]), Laos (10.1–30.4% [22]), China (9.3–42.9%) [20,21], and Malaysia (13.5–13.8% [24]). The *Bartonella* spp. prevalence in this study (34% Myanmar, 16% Sri Lanka) are in agreement with the previously mentioned studies [5].

The prevalence of Bartonella in small mammals from Sri Lanka was significantly lower than in Myanmar. In Sri Lanka, the cosmopolitan species *R. rattus* was introduced via ship trading to this island [53]. A possible explanation for the significantly lower prevalence in Sri Lanka could be first of all the absence of some parasites, which function as vectors (e.g., *Xenopsylla cheopis*) [5]. Moreover, ecological conditions on an island may limit the reproduction of the arthropod vector as it is the case in the Canary Islands. The study showed major variants of the *Bartonella* spp. prevalence in rodents at different islands. An explanation for this distribution was the varying ecological condition affecting the flea populations as essential vectors for the infection of rodents [54]. Various potential vectors are known for *Bartonella* spp.; however, the interaction between host and vector has not been clarified, yet [55]. The missing connection to continental rat populations may suppress reintroduction of suitable arthropod hosts and it can affect the presence and population size of competent rodent hosts in Sri Lanka but not in Myanmar [5].

Statistical analyses revealed significant differences in *Bartonella* spp. prevalence among small mammal species in Myanmar. Bandicoot rats had the highest (63%) and *S. murinus* the lowest prevalence (4%). In a recent study in Nepal, a low prevalence of *Bartonella* spp. was detected in *B. bengalensis* (26.3%) and high prevalence in *S. murinus* (64.1%) while *R. rattus* (43.3%) was intermediate. In Bangladesh both *B. bengalensis* (63.2%) and *S. murinus* (42.9%) showed higher prevalence than *R. rattus* [19,23]. The low prevalence of *S. murinus* might be due to a lack of host-specific vectors in Sri Lanka in our study. However, information about the ectoparasite fauna of *S. murinus* is lacking. Additionally, low Bartonella prevalence was detected in other insectivores (*Sorex araneus*) in England [56]. Investigations of *S. araneus* showed that some blood-sucking arthropods harboring rare *Bartonella* spp. may feed and transmit these *Bartonella* spp. exclusively to shrews. Furthermore, *S. araneus* was found to be less affected by ectoparasites compared to endemic rodents [56]. Therefore, the high prevalence of *S. murinus* may be related to an adapted ectoparasite fauna in Bangladesh but not in Myanmar or Sri Lanka. In Sri Lanka, the predominant rodent species *R. rattus* showed the highest prevalence (18%)—apart from the single *B. bengalensis*, which also tested positive. The prevalence for *R. rattus* and the high prevalence of *B. bengalensis* in Myanmar are in line with *R. rattus* in Asia (Malaysia: 13.5% [24], Laos: 10.1% [22], Taiwan: 10% [57]).

In Myanmar, the prevalence was higher in urban sites (Pike Kye We and Dayēbo) compared to the three rural sites. In line with our study, other authors reported higher prevalence of *Bartonella* spp. in small mammals from urban regions [58,59,60]. A possible explanation could be better conditions in cities for commensal rodents and thus for blood-sucking arthropods that serve as vectors for *Bartonella* spp., which can infect several mammals [5]. However, no significant difference in the prevalence of *Bartonella* spp. in Sri Lanka was detected concerning the different sites. All small mammals were caught in small-holder rice storage facilities in Sri Lanka and thus conditions were not as contrasting as at the sites in Myanmar.

In Myanmar, the prevalence in adult small mammals showed significantly higher prevalence than in subadults, possibly due to the life cycle of *Bartonella* spp. These bacteria can persist in the reservoir host without harming them drastically [61]. In contrast, Kosoy et al. [62] reported an increase in prevalence during the development of rodents especially from juveniles to subadults and detected lower prevalence in adult cotton rats (*Sigmodon hispidus*). The decrease of prevalence may be due to the development of immunity with age [62]. No differences in Bartonella prevalence according to age were detected in Sri Lanka. This might have been caused by the different small mammal species compositions. In Myanmar, high prevalence of *Bartonella* spp. was detected in *B. bengalensis* and *B. indica* and in Sri Lanka especially in *R. rattus*. Paziewska et al. [63] showed that the course of Bartonella infections depends on specific host-pathogen interactions. *Myodes glareolus* overcomes Bartonella infections within 1–2 months, however the length of time it takes to overcome the infection in *Apodemus flavicollis* is not definitely known. Furthermore, reinfections in *M. glareolus* were detected less frequently than in *A. flavicollis* [63]. Thus, Bartonella infections may persist in *Bandicota* spp. (more often collected in Myanmar) longer than in *Rattus* spp. (more often collected in Sri Lanka), which would explain the contrasting results in both countries.

In the present study, there was no effect of the seasons. Morway et al. showed that Bartonella prevalence rates were influenced by climatic conditions [64]. Moreover, a study detected higher Bartonella prevalence in rodents in Cambodia, Lao PDR, and Thailand during the wet season, because at that time there may be better living conditions for the vectors [65]. The lack of an effect of season may have been caused by the different composition of the small mammal community in our study during the year. Our results are in line with Kosoy et al. and others who showed no significant differences in Bartonella prevalence according to the small mammals’ sex [62]. No significant effect of sex was detected in other parts of Southeast Asia as well [65].

Kosoy et al. [66] showed that rodents are infected with different *Bartonella* spp. strains for a long time and that single strains replace each other. This infection strategy may have an impact on the probability of double infections in small mammals [66]. However, it is also known that reservoir hosts like rodents tolerate *Bartonella* spp. infections with more than one species and related *Bartonella* strains [67]. This diversity was explained by a lacking immune response of the reservoir hosts. Studies of Bartonella co-infections in small mammals are rare. In the current study, double infections were observed in 30 small mammals (18%) in Myanmar and 11 small mammals (14%) in Sri Lanka. We identified double infections in *B. bengalensis*, *R. rattus,* and *R. exulans* in Myanmar and exclusively in *R. rattus* in Sri Lanka at all sites. High co-infection rates (56%) with two or more genotypes, including recombinant variants, were detected in gerbils and fleas from Israel [68]. In Taiwan, a high prevalence was found in fleas and lice (64.7–64.9%), while a lower prevalence was found in small mammals (31.4%). Therefore, the sequencing results support the thesis of vectors bearing more different *Bartonella* spp. than their hosts [69]. In Southeast Asia, multiple infections are only reported in cats showing lower prevalence compared to our results (Thailand: 5.3–7.69%, China: 4.34%) [70,71,72].

Our sequence analysis of Bartonella-positive samples identified 11 different Bartonella strains. Moreover, *B. kosoyi,* first described in 2019, was the predominant species for the samples from Myanmar. Analysis confirmed a phylogenetic association of *B. kosoyi* with the zoonotic *B. grahamii*, *B. tribocorum*, and *B. elizabethae* [1]. *Bartonella elizabethae* and *B. elizabethae*-like species show host-specificity for rodents of the genus *Rattus* but can infect other small mammals like Bandicoot rats and *S. murinus* as well [5]. We detected *B. kosoyi* mainly in Bandicoot rats (*B. bengalensis*: 21/29, *B. indica*: 6/6). Before the Tel Aviv strain was named *B. kosoyi* sp. nov., this strain had been detected in Bandicoot rats and *R. rattus* from Bangladesh [19], Portugal [73] and Nepal [23]. Further, the Tel Aviv Strain was associated with a case of human lymphadenopathy and fever in Georgia [74]. Therefore, the zoonotic potential seems likely for *B. kosoyi*.

Some of the sequencing results from Myanmar were highly similar (99.81–100%) to *Bartonella* sp. SE-Bart-D isolated from the Oriental rat flea (*X. cheopis)* in Egypt. *Bartonella* sp. SE-Bart-D shared 85–90% similarity with *B. tribocorum*, *B. grahamii,* and *B. elizabethae,* which are all known as zoonotic agents [75].

In Sri Lanka, *Bartonella phoceensis* was the predominant result for all sequenced samples. *Bartonella phoceensis* was first detected along with *Bartonella rattimassiliensis* in France in 2004 [76]. Since then, *B. phoceensis* was detected in small mammals from Asia. In Japan and Thailand *B. phoceensis* was detected in *R. rattus* [67,77] and in Indonesia in *S. murinus* and *Rattus tanezumi* [78]. Moreover, *B. phoceensis* was associated, with a similarity of 94%, with uncultured Bartonella species found in *Rattus norvegicus* from Taiwan [57]. Until now, *B. phoceensis* is not recognized as a zoonotic agent and no case of human illness has been described.

*Bartonella henselae* was detected in one *S. murinus* from Myanmar in the current study. The organism with the highest similarity *B. henselae* Q5BJ-CW was first isolated from a dog in China. *Bartonella henselae* is known to be mostly transmitted by wild and domestic cats to humans [79]. Cat scratch disease caused by *B. henselae*, is associated with fever and regional lymphadenopathy [7]. Though not being considered as the main reservoir, rodents were reported as carriers of *B. henselae* in Italy and New Zealand. Therefore, rodents can be a source of infection due to close contact to humans [4,80]. Due to unspecific symptoms and neglected awareness, *Bartonella* spp. can be an underestimated threat to public health. Although the number of zoonotic *Bartonella* spp. has emerged, bartonellosis in humans is reported very rarely [3].

## 5. Conclusions

*Bartonella* spp. were detected for the first time in small mammals from Myanmar (34%) and Sri Lanka (16%) in this study. The high prevalence is in line with previously reported results from other countries in Southeast Asia. The reasons for the lower prevalence in Sri Lanka may be missing vectors or different ecological conditions. All examined small mammal species were Bartonella-positive in Myanmar and Sri Lanka. Eleven different *Bartonella* strains were detected of which three are considered potentially zoonotic. Thus, small mammals may serve as reservoir hosts for zoonotic *Bartonella* spp. in these countries. In Myanmar and Sri Lanka, contact between humans and rodents is frequent in rice storage and processing facilities. With an increasing degree of urbanization and thus more frequent contact between humans and rodents, these rodent-associated pathogens may pose a higher risk for human health.

## Figures and Tables

**Table 1:** Number of small mammal species collected at different study sites from Myanmar.

Small Mammal Species	No. of Collected Rodents	Trapping Location																			
		Dayēbo				PikeKye We				Kan Nyi Naung				Pha Aung We				Kadoke Phayargyi			
		Sex		Age		Sex		Age		Sex		Age		Sex		Age		Sex		Age	
		Male	Female	Sub-Adult	Adult	Male	Female	Sub-Adult	Adult	Male	Female	Sub-Adult	Adult	Male	Female	Sub-Adult	Adult	Male	Female	Sub-Adult	Adult
*Bandicota bengalensis*	155	36	27	-	63	39	36	4	71	2	3	3	2	2	3	1	4	4	3	-	7
*Bandicota indica*	8	4	-	-	4	3	-	-	3	-	-	-	-	-	-	-	-	1	-	-	1
*Rattus rattus*	72	2	-	1	1	-	-	-	-	15	29	24	20	2	9	4	7	6	9	7	8
*Rattus exulans*	150	6	8	14	-	5	10	13	2	24	34	49	9	10	31	36	5	7	15	20	2
*Suncus murinus*	110	9	12	4	17	6	5	3	8	1	2	1	2	33	30	22	41	8	4	4	8

No.: number; -: not detected.

**Table 2:** Number of small mammal species collected at different study sites from Sri Lanka.

Small Mammal Species	No. of CollectedRodents	Trapping Location																			
		Pasyala i				Pasyala ii				Kahapathwala i				Kahapathwala ii				Polonnaruwa i			
		Sex		Age		Sex		Age		Sex		Age		Sex		Age		Sex		Age	
		Male	Female	Sub-Adult	Adult	Male	Female	Sub-Adult	Adult	Male	Female	Sub-Adult	Adult	Male	Female	Sub-Adult	Adult	Male	Female	Sub-Adult	Adult
*Bandicota bengalensis*	1	-	-	-	-	-	-	-	-	-	-	-	-	-	1	-	1	-	-	-	-
*Bandicota indica*	13	2	2	2	2	2	3	1	4	1	1	1	1	1	-	-	1	1	-	-	1
*Rattus rattus*	433	37	47	48	36	30	56	58	28	35	54	44	45	36	52	54	34	29	57	57	29
*Rattus exulans*	5	1	1	2	-	-	-	-	-	-	-	-	-	-	-	-	-	-	3	3	-
*Suncus murinus*	43	3	4	7	-	2	5	6	1	5	4	9	-	3	7	8	2	3	7	10	-
*Mus booduga*	4	2	-	2	-	1	1	2	-	-	-	-	-	-	-	-	-	-	-	-	-

No.: number; -: not detected.

**Table 3:** Prevalence of *Bartonella* spp. DNA in small mammals from Sri Lanka and Myanmar targeting the *nuoG*, *gltA* gene and the 16s rRNA 23s rRNA ITS.

Country	Small Mammal Species	No. of Collected Small Mammals	No. of Samples Positive Targeting the *nuoG* Gene, the *gltA* Gene and ITS (No.; % (95% Cl))	No. of Samples Positive Targeting the *gltA* Gene and ITS (No.; % (95% Cl))	No. of Samples Positive Targeting ITS (No.; % (95% Cl))
Myanmar	*Bandicota bengalensis*	155	96; 61.94% (53.8–69.61)	99; 63.87% (55.78–71.42)	101; 65.16% (57.1–72.63)
	*Bandicota indica*	8	6; 75% (34.91–96.81)	6; 75% (34.91–96.81)	6; 75% (34.91–96.81)
	*Rattus rattus*	72	30; 41.67% (30.15–53.89)	30; 41.67% (30.15–53.89)	31; 43.06% (31.43–55.27)
	*Rattus exulans*	150	32; 21.33% (15.07–28.76)	42; 28% (20.98–35.91)	43; 28.67% (21.59–36.61)
	*Suncus murinus*	110	4; 3.64% (1–9.05)	9; 8.18% (3.81–14.96)	10; 9.09% (4.45–16.08)
	total	495	168; 33.94%;(29.77–38.3)	186; 37.58% (33.29–42.01)	191; 38.59% (34.28–43.03)
Sri Lanka	*Bandicota bengalensis*	1	1; 100%	1; 100%	1; 100%
	*Bandicota indica*	13	0; 0%	0; 0%	0; 0%
	*Rattus rattus*	433	79; 18.24% (14.72–22.21)	86; 19.86% (16.21–23.94)	90; 20.79% (17.06–24.92)
	*Rattus exulans*	5	0; 0%	0; 0%	0; 0%
	*Suncus murinus*	43	0; 0%	0; 0%	0; 0%
	*Mus booduga*	4	0; 0%	0; 0%	0; 0%
	total	499	80; 16.03% (12.92–19.55)	87; 17.43% (14.21–21.05)	91; 18.24% (14.94–21.91)

No.: number; CI: confidence interval.

**Table 4:** Sequencing results of 125 previously selected Bartonella-positive samples from Myanmar and Sri Lanka.

*Bartonella* Strains with the Highest Similarity in GenBank	GenBank ID with the Highest Similarity	Range of Similarity of the Detected Sequences (in %)	No. of Positive Individuals in This Study (Number of Sequences per Small Mammal Species)	Country in This Study
*Bartonella kosoyi*	CP031843	98.39–100%	41 (21 *Bandicota bengalensis*, 6 *Bandicota indica*, 4 *Rattus rattus*, 10 *Rattus exulans*)	Myanmar
		99.84–100%	3 (*Rattus rattus*)	Sri Lanka
*Bartonella phoceensis*	AY515123	99.8%	1 (*Rattus rattus*)	Myanmar
		99.8–100%	18 (*Rattus rattus*)	Sri Lanka
	MT792313	97.0–100%	7 (*Rattus rattus*)	Sri Lanka
*Bartonella henselae* Q5BJ-CW	JQ009430	99.83%	1 (*Suncus murinus*)	Myanmar
*Bartonella* sp. KM2529	EF202170	97.73–100%	11 (6 *Bandicota bengalensis*, 5 *Rattus exulans*)	Myanmar
*Bartonella* sp. KM2581	FJ667566	98.84–100%	14 (*Rattus rattus*)	Sri Lanka
*Bartonella* sp. SE-Bart-D	DQ166944	99.81–100%	9 (7 *Rattus exulans*, 1 *Rattus rattus*, 1 *Bandicota bengalensis*)	Myanmar
*Bartonella* sp. RN24BJ	EF190333	98.71%	1 (*Rattus exulans*)	Myanmar
*Bartonella* sp. RN25BJ	EF407566	99.16–100%	6 (3 *Rattus exulans*, 2 *Rattus rattus*, 1 *Bandicota bengalensis*)	Myanmar
*Bartonella* sp. RN28BJ	EF213776	97.53%	1 (*Rattus exulans*)	Myanmar
*Bartonella* sp. Rt222sm	AY277896	99.15%	1 (*Rattus rattus*)	Myanmar
uncultured *Bartonella* clone 2	MT271771	98.83%	1 (*Rattus exulans*)	Myanmar
Sequences of groups not considered *Bartonella*-positive due to low similarity levels:				
*Bartonella* sp. KM2529	EF202170	96.64%	Group 1 *: 3 (1 *Bandicota bengalensis*, 1 *Rattus rattus*, 1 *Rattus exulans*)	Myanmar
*Bartonella* sp. SE-Bart-D	DQ166944	89.98%	Group 2 *: 4 (*Rattus rattus*)	Sri Lanka
uncultured *Bartonella* clone 5199	MN244666	88.07%	Group 3 *: 3 (*Suncus murinus*)	Myanmar

No.: number; * these samples had a similarity of 98–100% compared to one another but yield not enough similarity to sequences in GenBank to be assigned to a certain *Bartonella* sp. sequence.

## Supplementary Materials

The following are available online at https://www.mdpi.com/2076-2607/9/3/658/s1, Table S1: Bartonella prevalence in rodents from Myanmar according to sex, age, year and season, Table S2: Bartonella prevalence in small mammals from Sri Lanka according to sex, age, year and season.
