# Supplementary material for: First Detection of Bartonella spp. in Small Mammals from Rice Storage and Processing Facilities in Myanmar and Sri Lanka
Source: Microorganisms. 2021 Mar 22;9(3):658. doi: 10.3390/microorganisms9030658 (PMC8004705; doi:10.3390/microorganisms9030658)
Supplement: Supplementary file 1 [file microorganisms-09-00658-s001.zip › Table S2.docx]

**Table S2:** *Bartonella* prevalence in small mammals from Sri Lanka according to sex, age, year and season

| **Biometric category of small mammals** | | ***Bartonella* spp. positive samples total (no; % (95% CI))** | ***Bartonella* spp. positive samples divided into sites (no; % (95% Cl)** | | | | |
| --- | --- | --- | --- | --- | --- | --- | --- |
|  |  |  | **Pasyala i** | **Pasyala ii** | **Kahapath- wala i** | **Kahapath- wala ii** | **Polonnaruwa i** |
| Sex | male | 30; 15.46% (10.68-21.33) | 7; 23.33% (9.93-42.28) | 6; 20%  (7.71-38.57) | 8; 26.67% (12.28-45.89) | 5; 16.67% (05.64-34.72) | 4; 13.33% (3.76-30.72) |
|  | female | 50; 16.39% (12.42-21.04) | 7; 14%  (5.82-26.74) | 11; 22% (11.53-35.96) | 15; 30% (17.86-44.61) | 8; 16%  (7.17-29.11) | 9; 18%  (8.58-31.44) |
| Age | sub-adult | 29; 9.35% (6.35-13.16) | 5; 17.24% (5.85-35.77) | 7; 24.14% (10.3-43.54) | 10; 34.48% (17.94-54.33) | 2; 6.9% (0.85-22.77) | 5; 17.24% (5.85-35.77) |
|  | adult | 51; 37.78% (29.58-46.52) | 9; 17.65%  (8.4-30.87) | 10; 19.61% (9.82-33.12) | 13; 25.49% (14.33-39.63) | 11; 21.57% (11.29-35.32) | 8; 15.69% (7.02-28.59) |
| Year | 2018 | 40; 16.06% (11.73-21.23) | 9; 22.5% (10.84-38.45) | 10; 25% (12.69-41.2) | 9; 22.5% (10.84-38.45) | 6; 15%  (5.71-29.84) | 6; 15%  (5.71-29.84) |
|  | 2019 | 40; 16%  (11.68-21.14) | 5; 12.5% (4.19-26.8) | 7; 17.5% (7.34-32.78) | 14; 35% (20.63-51.68) | 7; 17.5% (7.34-32.78) | 7; 17.5%  (7.34-32.78) |
| Season | dry | 61; 15.97% (12.44-20.03) | 12; 19.67% (10.6-31.84) | 11; 18.03% (9.36-29.98) | 16; 26.23% (15.8-39.07) | 9; 14.75% (6.98-26.17) | 13; 21.31% 11.86-33.68) |
|  | wet | 19; 16.24% (10.07-24.19) | 2; 10.53% (1.3-33.14) | 6; 31.58% (12.58-56.5) | 7; 36.84% (16.29-61.6) | 4; 21.05% (6.05-45.57) | - |

No.: number; ‘-‘: not detected; CI: confidence interval
