# Supplementary material for: First Detection of Bartonella spp. in Small Mammals from Rice Storage and Processing Facilities in Myanmar and Sri Lanka
Source: Microorganisms. 2021 Mar 22;9(3):658. doi: 10.3390/microorganisms9030658 (PMC8004705; doi:10.3390/microorganisms9030658)
Supplement: Supplementary file 1 [file microorganisms-09-00658-s001.zip › Table S1 MDPI.docx]

**Table S1**: *Bartonella* prevalence in rodents from Myanmar according to sex, age, year and season

| **Biometric category of small mammals** | | ***Bartonella* spp. positive samples total (no; % (95% CI))** | ***Bartonella* spp. positive samples divided into trapping sites (no; % (95% Cl))** | | | | |
| --- | --- | --- | --- | --- | --- | --- | --- |
|  |  |  | **Dayēbo** | **Pike Kye We** | **Kan Nyi Naung** | **Pha Aung We** | **Kadoke Phayargyi** |
| Sex | male | 79; 35.11%  (28.89-41.73) | 32; 40.51% (29.6-52.15) | 30; 37.97% (27.28-49.59) | 9; 11.39%  (5.34-20.53) | 3; 3.8%  (0.79-10.7) | 5; 6.33%  (2.09-14.16) |
|  | female | 89; 32.96%  (27.39-38.92) | 13; 14.61% (8.01-23.68) | 29; 32.58% (23.02-43.34) | 26; 29.21% (20.05-39.81) | 12; 13.48% (7.17-22.37) | 9; 10.11% (4.73-18.33) |
| Age | sub-adult | 33; 15.71% (11.07-21.35) | 1; 3.03% (0.08-15.76) | 3; 9.09% (1.92-24.33) | 17; 51.52% (33.54-69.2) | 5; 15.15% (5.11-31.9) | 7; 21.21% (8.98-38.91) |
|  | adult | 135; 47.37% (41.45-53.35) | 44; 32.59% (24.78-41.2) | 56; 41.48% (33.07-50.27) | 18; 13.33% (8.1-20.25) | 10; 7.41% (3.61-13.2) | 7; 5.19% (2.11-10.39) |
| Year | 2018 | 90; 36.59%  (30.56-42.94) | 28; 31.11% (21.77-41.74) | 26; 28.89% (19.82-39.4) | 27; 30%  (20.79-40.57) | 3; 3.33%  (0.69-9.43) | 6; 6.67%  (2.49-13.95) |
|  | 2019 | 78; 31.33%  (25.62-37.48) | 17; 21.79% (13.24-32.59) | 33; 42.31% (31.19-54.02) | 8; 10.26%  (4.53-19.21) | 12; 15.38% (8.21-25.33) | 8; 10.26%  (4.53-19.21) |
| Season | dry | 57; 33.73% (26.65-41.39) | 10; 17.54% (8.75-29.91) | 14; 24.56% (14.1-37.76) | 17; 29.82% (18.43-43.4) | 9; 15.79% 7.48-27.87) | 7; 12.28% (5.08-23.68) |
|  | wet | 111; 34.05%  (28.92-39.47) | 35; 31.53% (23.1-41.04) | 45; 40.54% (31.3-50.27) | 18; 16.22% (9.9-24.41) | 6; 5.41% (2.01-11.39) | 7; 6.31% (2.57-12.56) |

No.: number; ‘-‘: not detected; CI: confidence interval
